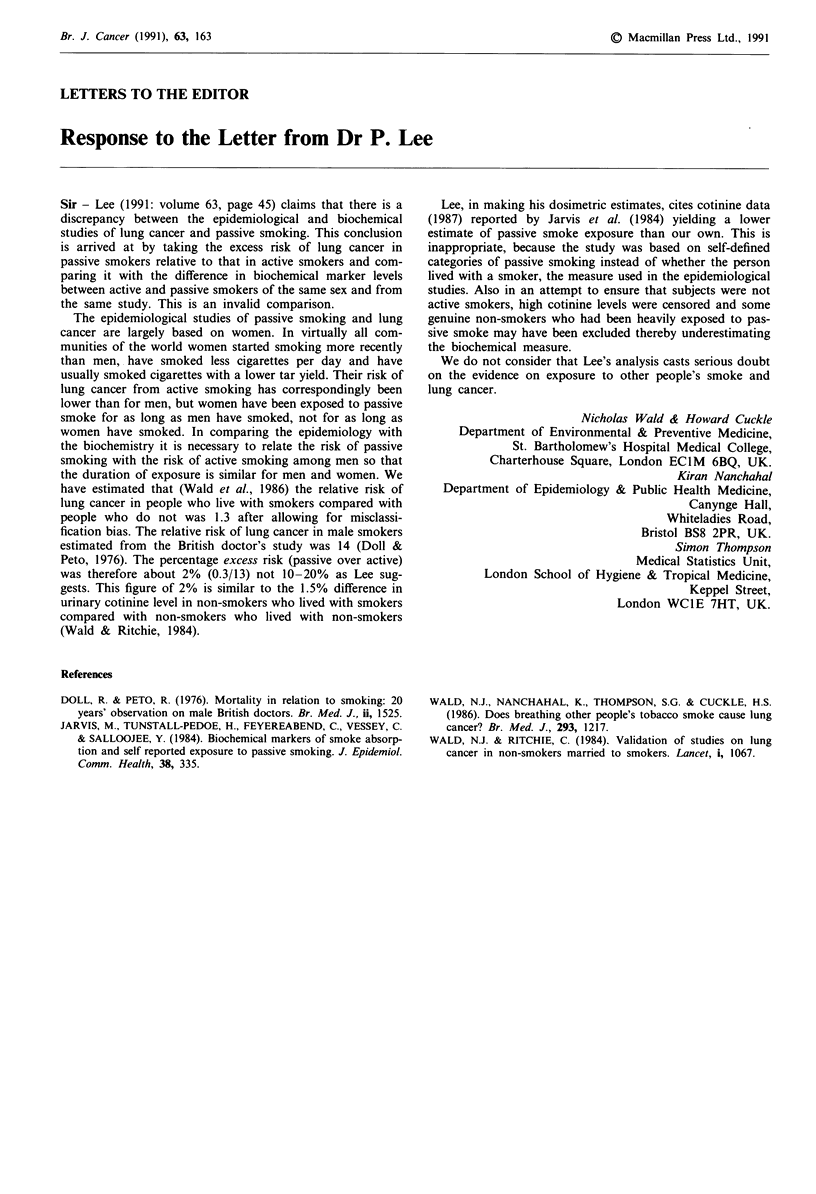# Respnose to the letter from Dr Lee

**Published:** 1991-01

**Authors:** Nicholas Wald, Howard Cuckle, Kiran Nanchahal, Simon Thompson


 © Macmillan Press Ltd., 1991

### LETTERS TO THE EDITOR

### Response to the Letter from Dr P. Lee

**Sir** – Lee (1991: volume 63, page 45) claims that there is a discrepancy between the epidemiological and biochemical studies of lung cancer and passive smoking. This conclusion is arrived at by taking the excess risk of lung cancer in passive smokers relative to that in active smokers and comparing it with the difference in biochemical marker levels between active and passive smokers of the same sex and from the same study. This is an invalid comparison.

The epidemiological studies of passive smoking and lung cancer are largely based on women. In virtually all communities of the world women started smoking more recently than men, have smoked less cigarettes per day and have usually smoked cigarettes with a lower tar yield. Their risk of lung cancer from active smoking has correspondingly been lower than for men, but women have been exposed to passive smoke for as long as men have smoked, not for as long as women have smoked. In comparing the epidemiology with the biochemistry it is necessary to relate the risk of passive smoking with the risk of active smoking among men so that the duration of exposure is similar for men and women. We have estimated that (Wald *et al.*, 1986) the relative risk of lung cancer in people who live with smokers compared with people who do not was 1.3 after allowing for misclassification bias. The relative risk of lung cancer in male smokers estimated from the British doctor's study was 14 (Doll & Peto, 1976). The percentage *excess* risk (passive over active) was therefore about 2% (0.3/13) not 10–20% as Lee suggests. This figure of 2% is similar to the 1.5% difference in urinary cotinine level in non-smokers who lived with smokers compared with non-smokers who lived with non-smokers (Wald & Ritchie, 1984).

Lee, in making his dosimetric estimates, cites cotinine data (1987) reported by Jarvis *et al.* (1984) yielding a lower estimate of passive smoke exposure than our own. This is inappropriate, because the study was based on self-defined categories of passive smoking instead of whether the person lived with a smoker, the measure used in the epidemiological studies. Also in an attempt to ensure that subjects were not active smokers, high cotinine levels were censored and some genuine non-smokers who had been heavily exposed to passive smoke may have been excluded thereby underestimating the biochemical measure.

We do not consider that Lee's analysis casts serious doubt on the evidence on exposure to other people's smoke and lung cancer.

*Nicholas Wald & Howard Cuckle*
Department of Environmental & Preventive Medicine,
St. Bartholomew's Hospital Medical College,
Charterhouse Square, London EC1M 6BQ, UK.
*Kiran Nanchahal*
Department of Epidemiology & Public Health Medicine,
Canynge Hall,
Whiteladies Road,
Bristol BS8 2PR, UK.
*Simon Thompson*
Medical Statistics Unit,
London School of Hygiene & Tropical Medicine,
Keppel Street,
London WC1E 7HT, UK.

**References**

DOLL, R. & PETO, R. (1976). Mortality in relation to smoking: 20 years' observation on male British doctors. *Br. Med. J.*, **ii**, 1525.

JARVIS, M., TUNSTALL-PEDOE, H., FEYEREABEND, C., VESSEY, C. & SALLOOJEE, Y. (1984). Biochemical markers of smoke absorption and self reported exposure to passive smoking. *J. Epidemiol. Comm. Health*, **38**, 335.

WALD, N.J., NANCHAHAL, K., THOMPSON, S.G. & CUCKLE, H.S. (1986). Does breathing other people's tobacco smoke cause lung cancer? *Br. Med. J.*, **293**, 1217.

WALD, N.J. & RITCHIE, C. (1984). Validation of studies on lung cancer in non-smokers married to smokers. *Lancet*, **i**, 1067.